# Bioprospection of biocompounds and dietary supplements of microalgae with immunostimulating activity: a comprehensive review

**DOI:** 10.7717/peerj.7685

**Published:** 2019-10-01

**Authors:** Arialdo M. Silveira Júnior, Silvia Maria M. Faustino, Alan C. Cunha

**Affiliations:** 1 Department of Environment and Development, Federal University of Amapá, Macapá, Amapá, Brazil; 2 Postgraduate Program in Tropical Biodiversity, Federal University of Amapá, Macapá, Amapá, Brazil; 3 Department of Biological and Health Sciences, Federal University of Amapá, Macapá, Amapá, Brazil; 4 Department of Exact and Natural Sciences, Federal University of Amapá, Macapá, Amapá, Brazil

**Keywords:** Biotechnology, Fish farming, Biodiversity, Immunostimulation, Brazilian Amazon, Biotec

## Abstract

The objective of this review is to analyze the role of microalgal bioprospecting and the application of microalgae as food supplements and immunostimulants in global and regional aquaculture, highlighting the Brazilian Amazon. This study evaluates the primary advantages of the application of the bioactive compounds of these microorganisms, simultaneously identifying the knowledge gaps that hinder their biotechnological and economic exploitation. The methodology used is comparative and descriptive-analytical, considering the hypothesis of the importance of bioprospecting microalgae, the mechanisms of crop development and its biotechnological and sustainable application. In this context, this review describes the primary applications of microalgae in aquaculture during the last decade (2005–2017). The positive effects of food replacement and/or complementation of microalgae on the diets of organisms, such as their influence on the reproduction rates, growth, and development of fish, mollusks and crustaceans are described and analyzed. In addition, the importance of physiological parameters and their association with the associated gene expression of immune responses in organisms supplemented with microalgae was demonstrated. Complementarily, the existence of technical-scientific gaps in a regional panorama was identified, despite the potential of microalgal cultivation in the Brazilian Amazon. In general, factors preventing the most immediate biotechnological applications in the use of microalgae in the region include the absence of applied research in the area. We conclude that the potential of these microorganisms has been relatively well exploited at the international level but not at the Amazon level. In the latter case, the biotechnological potential still depends on a series of crucial steps that involve the identification of species, the understanding of their functional characteristics and their applicability in the biotechnological area, especially in aquaculture.

## Introduction

Microalgae are unicellular or colonial photosynthetic organisms that are primarily found in natural aquatic environments, such as inland waters and coastal sea areas. There are estimated to be between 200,000 and 800,000 species of microalgae worldwide (Ratha & Prasanna, 2012), and they present great potential for use in biotechnology, biorefinery and bioprospection applications (Brasil, Silva & Siqueira, 2017).

Bioprospecting is understood as the use of biodiversity in the provision of resources for the discovery, classification, investigation and/or formulation of new sources of chemical compounds, genes, proteins, and other components with potential economic and biotechnological value (Sacarro Junior, 2011; Berlinck, 2012; Marques et al., 2013).

Overall, the use of macro- and microalgae for bioprospecting has substantially increased in recent decades, generating a biomass market with annual values between US $ 3.8 and 5.4 billion (Brasil, Silva & Siqueira, 2017) and involving the pharmaceutical, veterinary, nutraceutical, biomedical, bioenergetic, food, and public health sectors (Marinho-Soriano et al., 2012). In addition, interest has increased in these microorganisms as a source of biologically active components for the formulation of products in animal feed, including aquaculture, generating 20.68% of the inputs used in patents for this purpose (Barcellos et al., 2012; Stranska-Zachariasova et al., 2016).

Aquaculture is rapidly developing worldwide, becoming the principal subsidiary of the fishing industry (Milhazes-Cunha & Otero, 2017; Grealis et al., 2017) and the fastest growing food industry in recent years (Han et al., 2017; Ansari et al., 2017). This growth has aroused scientific and biotechnological interest in the improvement and maintenance of aquaculture processes, including the use of microalgae in animal feed, which has been discussed in recent decades.

The use of microalgae has shown promising results in aquaculture, with positive effects observed with respect to food digestibility and the growth and survival of organisms, as well as on gene expression and immune responses in assayed fish, mollusks and crustaceans (Cerezuela et al., 2012; Carboni et al., 2012; Ju, Deng & Dominy, 2012; Reyes-Becerril et al., 2014; Zhang et al., 2014; Chen, Zhao & Qi, 2015; Arney et al., 2015; Adel et al., 2016; Barron et al., 2016; Vizcaíno et al., 2016; Tibbetts, Yasumaru & Lemos, 2017). In addition, microalgae are essential sources of nutrients and proteins in animal feed (Araujo et al., 2011), which in aquaculture can account for up to 80% of production costs, where protein sources are the most costly ingredients related to diet (Sandre et al., 2017).

The most widely used microalgae in aquaculture are *Chlorella*, *Tetraselmis*, *Isochrysis*, *Pavlova*, *Phaeodactylum*, *Chaetoceros*, *Nannochloropsis*, *Skeletonema* and *Thalassiosira*, and combinations of different species can provide an adequate balance of proteins, lipids and micronutrients essential for the development of cultivation of organisms (Charoonnart, Purton & Saksmerprome, 2018). This result stems from the significant ability of microalgae to convert atmospheric CO_2_ to useful products such as carbohydrates, lipids and other bioactive compounds (Khan, Shin & Kim, 2018). Furthermore, microalgae provide a good possibility of being genetically modified for desired metabolic traits (Johanningmeier & Fischer, 2010; Gimpel, Henríquez & Mayfield, 2015), broadening its uses and efficiency.

Regionally, the Brazilian Amazon is responsible for more than half of Brazil’s fishery production in inland waters. However, Brazil’s natural stocks have been suffering from predatory fishing, especially in rivers with low nutrient load, and consequently, lower productivity (Viana, 2013). This loss has led to the need to develop more effective techniques for aquaculture, encouraging the cultivation of different species in this region, especially fish and crustaceans.

In addition, the Brazilian Amazon, together with other Brazilian ecosystems, contributes approximately 25% of the microalgae found worldwide (Agostinho, Thomaz & Gomes, 2005; Cunha et al., 2013; Silveira Júnior et al., 2015), with ≈3,500 species of microalgae cataloged (Brasil, Silva & Siqueira, 2017). However, paradoxically, bioprospecting and biotechnology processes are far from reflecting the potential of Brazil’s megabiodiversity. Historically, the natural resources of Brazil have been poorly explored (Mesquita et al., 2015), with evidence indicating a lack of studies on microalgae as well as of basic knowledge regarding the potential for the sustainable exploitation of microalgae.

This review presents a comparative and descriptive-analytical analysis of the latest advances in microalgal bioprospecting and its application in global aquaculture, with an emphasis on the Amazon region. In addition, the importance of the microalgal bioprospecting processes are discussed, especially with respect to the synthesis of bioactive compounds and their potential applications in food supplementation and immunostimulation for aquaculture.

### Survey methodology

This review is characterized by an extensive collection and compilation of scattered data in the literature (journals from databases such as Web of Science, ScienceDirect, SciELO, Scopus and PubMed, including theses and thematic dissertations), where the selection criteria primarily involved the adequacy of the data to the thematic proposal of this review. The search terms that were used when searching for articles included microalgae, aquaculture, immunostimulating, food supplementation in fish, fish farming and Amazon. Because the temporal analysis included databases from the last decade, Tables were generated related to the production of bioactive compounds as well as food supplementation and immunostimulation of aquatic organisms in global and regional aquaculture processes.

### Bioprospecting and the cultivation of microalgae

Large-scale microalgal cultivation began to be developed in the middle of the last century, leading to numerous commercial applications and biotechnological interests (Pringsheim, 1912; Harun et al., 2010; Stranska-Zachariasova et al., 2016). The primary objective of cultivating these crops has been to obtain biomass for the generation of inputs used for different purposes, primarily as renewable energy resources (Posten, 2009; Hempel, Petrick & Behrendt, 2012; Adams et al., 2013; Wen et al., 2016; Mallick et al., 2016), the production of biomolecules (β-carotene and astaxanthin) and biocolorants, wastewater treatment, bioremediation and use in aquaculture (Ansari et al., 2017).

Because they are photosynthetic organisms with simple nutritional requirements, the production of microalgal biomass is easily employed and has great potential for the obtainment of biocompounds (Andrade & Costa, 2008; Posten, 2009; Hempel, Petrick & Behrendt, 2012; Adams et al., 2013; Wen et al., 2016; Mallick et al., 2016). However, despite their rapid growth, high lipid content (Tan & Lee, 2016; Wang, Sheng & Yang, 2017), environmental impact mitigation efficiency, noncompetition with agricultural land crops (Mallick et al., 2016) and lower water demand than terrestrial crops (Zhu, Li & Hiltunen, 2016; Tan & Lee, 2016), there are difficulties in microalgal cultivation for biotechnology purposes.

These difficulties are directly related to the economic feasibility of the processes used for culturing microalgae and the final obtainment of biomass. For example, the separation of biomass and the extraction of important biocompounds from bioprospecting processes can represent from 3.3 to 30% of the total cost of production, depending on the species and type of culture used (open or closed).

Therefore, the commercial production of microalgae must overcome critical problems related to its economic viability and the high operational costs of cultivation and processing (Calixto et al., 2016). Laboratory and semi-industrial scale cultivation has already been well studied, but this level of study is not the case for large-scale cultivation, either in open or closed systems (Abo et al., 2019).

Open microalgae productivity (open system) results in a dry weights of 20–40 g m^2^ day^−1^ biomass and a maximum solar conversion efficiency of 3 to 10%, which is 10 to 50 times greater than the efficiency exhibited by terrestrial plants (Hempel, Petrick & Behrendt, 2012; Chen, Zhao & Qi, 2015; Wen et al., 2016; Mallick et al., 2016; Mohammadi, Arabian & Khalilzadeh, 2016; Tan & Lee, 2016). This productivity increases when evaluations are performed under laboratory conditions (closed system) (Wen et al., 2016), suggesting an interesting advantage to its production on an industrial scale. However, the high cost for the production of this last type of culture, which involves the use of bioreactors, and its associated production costs (Das et al., 2015; Mohammadi, Arabian & Khalilzadeh, 2016) makes this activity still economically unviable (Guo et al., 2013; Jebali et al., 2015).

Efforts have been made to improve the cost-effectiveness of microalgal cultivation, such as the genetic improvement of strains for a combination of high productivity and the adequate synthesis of compounds that are useful for bioprospecting and biotechnology (Dao et al., 2018). However, only approximately 20 species of different microalgae, including cyanobacteria, have been successfully genetically modified to date, mostly in studies with the species *Chlamydomonas reinhardtii* (Benedetti et al., 2018; Spicer & Molnar, 2018).

Although genetic modifications may be financially profitable, it is notable that the research, development, adoption of genetically modified strains and regulatory requirements can be quite expensive and requires significant initial capital investment. For example, for the production of genetically engineered algae, stricter regulations would require indoor cultivation under artificial lighting in a closed and contained system, significantly adding to the costs (Charoonnart, Purton & Saksmerprome, 2018).

Even with these barriers, many species of microalgae are used for the bioprospecting of bioactive compounds (vitamins, pigments, fatty acids, amino acids, and carbohydrates). Currently, the most relevant species for the production of these compounds with high value in use are the cyanobacteria *Arthrospira platensis* and the green microalgae *Chlorella vulgaris*, *Dunaliella salina* and *Haematococcus pluvialis*, with the last two in large agricultural systems for the production of carotenoids (Benedetti et al., 2018).

Moreover, there have been increases in the production of biomass grown in both open ponds and photobioreactor systems (Posten, 2009; Wen et al., 2016) that has resulted in a large quantity of research in the last five years with respect to the use of this biomass as a dietary supplement for aquaculture (Cerezuela et al., 2012; Carboni et al., 2012; Ju, Deng & Dominy, 2012; Reyes-Becerril et al., 2013; Reyes-Becerril et al., 2014; Zhang et al., 2014; Chen, Zhao & Qi, 2015; Arney et al., 2015; Adel et al., 2016; Vizcaíno et al., 2016; Barron et al., 2016; Tibbetts, Yasumaru & Lemos, 2017).

Thus, the advances in the biotechnological use of microalgae have shown compelling results in the global literature, primarily due to the microalgal accumulation of important biocomponents, such as lipids (fatty acids), proteins and polysaccharides (carbohydrates) (Dao et al., 2018). In addition, the yield of microalgal cultivation (growth rate and biomass production) may be substantial (Fré, 2016), showing an economic relevance (potential) for bioprospecting from this input.

### Production of bioactive compounds by microalgae and their potential use in aquaculture

Microalgae are a source of a wide and unpredictable range of compounds (Derner et al., 2006), such as pigments, oils, hydrocarbons, carbohydrates and proteins that can make products of variable nature and that are produced in different ratios (Angelo, Andrade & Colozzi Filho, 2014; Mallick et al., 2016). Alternative culture media are tested to increase this productivity (Baumgartner et al., 2013), with emphasis on the use of sterilized domestic sewage (Chen, Zhao & Qi, 2015), biodigester effluents, digested sludge, sugarcane vinasse, wastewater from olive oil production, swine farming effluent (Andrade & Costa, 2008; Bertoldi, Sant’Anna & Oliveira, 2008) and aquaculture wastewater (Guo et al., 2013; Gao et al., 2016).

The nutritional source of the culture is the primary influencer of the intracellular synthesis of microalgae (Mohammadi, Arabian & Khalilzadeh, 2016; Bekirogullari et al., 2017), and a deficit or excess of nutrients influences both the lipid contents and the synthesis of other bioactive compounds that ensure the survival of cells in culture (Adams et al., 2013; Zhu, Li & Hiltunen, 2016; Mallick et al., 2016). In addition, abiotic stress due to luminosity, nutritional restriction and thermal changes are also variables that are related to this synthesis (Radmann & Costa, 2008; Baumgartner et al., 2013; Mohammadi, Arabian & Khalilzadeh, 2016).

The high contents of macro- and micronutrients present in the biomass, along with the protein content, amino acid profile and the presence of fatty acids, make this raw material promising for its incorporation in the diet of aquatic organisms, especially in the initial phase of their life cycle (Vizcaíno et al., 2016). The high content of compounds present in the metabolism of microalgae, together with their high growth rate and yield, increases the interest in the use of these organisms for aquaculture (Freire et al., 2016) (Table 1).

**Table 1:** Growth rate and protein, carbohydrate and lipid contents in microalgae grown in studies reported in the literature by geographical area. (a) values in mg L^−1^; (b) values in g.L^−1^.d^−1^; (c) values in %; (d) values in µg mL^−1^ ± SD. (*) approximated values.

**Microalgae**	**Growth rate (µ**_**max**_**)**	**Protein(mean ± SD or %)**	**Carbohydrate(mean ± SD or %)**	**Lipid contents (%)**	**Geographical area**	**References**
*Chlamydomonas reinhardtii*	0.0094 d^−1^			26c	Great Britain	Bekirogullari et al. (2017)
*Arthrospira platensis*	0.266 d^−1^		0.116 ± 0.002b		Brazil	Margarites (2014)
*Arthrospira platensis*		37.7c			Brazil	Pelizer, Carvalho & Moraes (2015)
*Arthrospira platensis*	0.12	72c			Brazil	Avila-Leon et al. (2012)
*Chlorella homosphaera*	0.104 d^−1^		0.014 ± 0,001b		Brazil	Margarites (2014)
*Chlorella minutíssima*		14.9 ± 1,3d	6.6 ± 0,3d	38c	Brazil	Borges-Campos, Barbarino & Lourenço (2010)
*Chlorella saccharophila*				27,6c	Germany	Hempel, Petrick & Behrendt (2012)
*Chlorella sorokiniana*				47c	USA	Adams et al. (2013)
*Chlorella sorokiniana*		36c	20c	19,8c	South Africa	Gupta et al. (2017)
*Chlorella* sp.	0.18 d^−1^			13c	India	Bruno, Udhaya & Sandhya (2013)
*Chlorella* sp.	0.495 d^−1^			30,2c	Germany	Hempel, Petrick & Behrendt (2012)
*Chlorella* sp*.*				48,9c	Lithuania	Makareviciene et al. (2011)
*Chlorella* sp.				49,7c	Thailand	Cheirsilp, Mandik & Prasertsan (2016)
*Chlorella vulgaris*				48c	USA	Adams et al. (2013)
*Chlorella vulgaris*				38c	USA	Liang, Sarkany & Cui (2009)
*Chlorella vulgaris*	0.573 d^−1^			12,2c	Iran	Mohammadi, Arabian & Khalilzadeh (2016)
*Chlorella vulgaris*		53,1 c	17,9c		Holland	Postma et al. (2017)
*Chlorella vulgaris*				5,21c	Brazil	Radmann & Costa (2008)
*Chlorococcum echinozygotum*	0.13 d^−1^			21c	India	Bruno, Udhaya & Sandhya (2013)
*Chlorococcum oleofaciens*				46c	USA	Adams et al. (2013)
*Chlorococcum oleofaciens*		35c*	51c*	39c*	Spain	Del Río et al. (2017)
*Coelastrum microporum*	0.29 d^−1^			29c	India	Bruno, Udhaya & Sandhya (2013)
*Dunaliella tertiolecta*		26.0 ± 1.3d	9.2 ± 0.5d	41,8c	Brazil	Brandão, Gomes & Chagas (2006)
*Graesiella* sp*.*		13c*	35c*	45,2c	China	Wen et al. (2016)
*Isochrysis galbana*		29.4 ± 1.9d	18.6 ± 1.7d	38,7c	Brazil	Borges-Campos, Barbarino & Lourenço (2010)
*Isochrysis* sp*.*	0.18 d^−1^			23,5c	Australia	Huerlimann, Nys & Heimann (2010)
*Nannochloropsis*		36,4c	12,4c	27,8c	China	Wang, Sheng & Yang (2017)
*Nannochloropsis* sp*.*	0.32 d^−1^			21,3c	Australia	Huerlimann, Nys & Heimann (2010)
*Neochloris oleoabundans*				58c	USA	Adams et al. (2013)
*Neochloris oleoabundans*		55,6c	17,1c		Holland	Postma et al. (2017)
*Phaeodactylum tricornutum*		23.3 ± 0.8d	13.1 ± 0.5d	35c	Brazil	Borges-Campos, Barbarino & Lourenço (2010)
*Pseudokirchneriella subcapitata*		40c*	30c*	46c*	Spain	Del Río et al. (2017)
*Rhodomonas* sp*.*	0.26 d^−1^			9,5c	Australia	Huerlimann, Nys & Heimann (2010)
*Scenedesmus pectinatus*	0.23 d^−1^			16c	India	Bruno, Udhaya & Sandhya (2013)
*Scenedesmus dimmorphus*				34c	USA	Adams et al. (2013)
*Scenedesmus naegelii*				39c*	USA	Adams et al. (2013)
*Scenedesmus obliquus*		37c	20.4c	16c	South Africa	Gupta & Ahmad (1966)
*Scenedesmus obliquus*				6,18c	Brazil	Radmann & Costa (2008)
*Scenedesmus obtusiusculus*		25.9c	50c	19,9c	Germany	Schulze et al. (2016)
*Scenedesmus* sp.			20c*	60c*	Italy	Di Caprio et al. (2016)
*Scenedesmus* sp*.*				51,9c	Lithuania	Makareviciene et al. (2011)
*Skeletonema costatum*		14.9 ± 0.8d	8.4 ± 0.4d	34,4c	Brazil	Borges-Campos, Barbarino & Lourenço (2010)
*Synechococcus nidulans*				5c	Brazil	Radmann & Costa (2008)
*Tetraselmis* sp*.*	0.19 d^−1^			10,6c	Australia	Margarites (2014)
*Tetraselmis suecica*		43.3c	21.2c		Holland	Postma et al. (2017)
*Trichosporon oleaginosus*				53c	Germany	Meo, Priebe & Weuster-Botz (2017)

Protein levels in microalgae are often greater than 30%, while lipid levels range from 5.21% to 60.7% (Table 1), both of which depend on the cultivated species and may vary according to the culture medium used (Tibaldi et al., 2015), influencing the net yield (biomass produced).

The rate of cell growth, ranging from 0.0094 day^−1^ to 0.573 day^−1^ (Table 1), and the production of quality biocompounds are important variables to determine the viability (cost reduction and efficiency) of bioprospecting microalgae (Dao et al., 2018). The balance between these factors (biomass production and intracellular synthesis) is crucial to achieve maximum productivity and the adequate production of metabolites, leading to studies being performed to improve crops to meet this demand and to make the biotechnological use of microalgae possible (Tsigie et al., 2012; Li et al., 2013; Sforza, Barbera & Bertucco, 2015; He, Yang & Hu, 2015).

The high protein content metabolized in microalgal species, which can reach 72% of its dry weight (Table 1), has led to its use as an unconventional protein source in the feeding of aquatic organisms (Spolaore et al., 2006). Species of the genera *Arthrospira* (Madeira et al., 2017) and *Chlorella* have been identified as valuable sources of proteins (D’Este, Alvarado-Morales & Angelidaki, 2017), with the latter species being isolated and cultivated primarily for the extraction of its bioactive compounds. These species have protein levels ranging from 36.0 to 72.0%, as shown in Table 1.

Likewise, some species of microalgae have a high lipid content (above 30%) (Harun et al., 2010) and are therefore recognized as an alternative source for the production lipid-containing compounds, with significant levels of production observed (between 5 and 60.7% of the dry weight) (Table 1). These species can often also be induced to produce different types of fatty acids by altering the temperature, pH and nutrient concentrations in the culture (Araujo et al., 2011). However, nutritional stress that leads to the substantial accumulation of cellular lipids may inhibit cell growth (biomass), resulting in low net oil yields (Bekirogullari et al., 2017) and making bioprospecting potentially unfeasible, which requires further conclusive studies.

The high content of fatty acids (lipids) present in the microalgal intracellular content promotes the development, survival and deposition of nutrients in aquatic organisms (Barcellos et al., 2012). The production of omega-3, omega-6 and polyunsaturated fatty acids is essential and promising for animal nutrition (Taelman et al., 2013; Ryckebosch et al., 2014), as is the production of carotenoids with antioxidant effects (Foo et al., 2017).

Among the fatty acids in the omega-3 family, eicosapentaenoic acid (EPA) and docosahexaenoic acid (DHA) (Ryckebosch et al., 2014) are essential in animal dietary supplementation, replacing conventional sources of oils, such as those from oily fish (Tsai, Chuang & Chen, 2016). In addition, proteins, lipids and carbohydrates, as well as vitamins, minerals and other bioactive compounds are important nutritional components in aquaculture (Ayadi, Rosentrater & Muthukumarappan, 2012; Madeira et al., 2017). Thus, microalgae have important characteristics for use as a natural supplement in animal feed to replace synthetic components or to meet growing aquaculture input demands (Yaakob et al., 2014).

Thus, the observed production of microalgae biomass and bioactive compounds with high nutritional value (Kiron et al., 2012) confirm their for use in biotechnological purposes (Barcellos et al., 2012; Zhang et al., 2014). These compounds, besides being useful in the development of functional foods due to their antioxidant properties (Taelman et al., 2013), also have the capacity to reduce side effects in the control of diseases and generate fewer environmental impacts when used in aquaculture (Adel et al., 2016). However, some reactive oxygen species (ROS), such as the superoxide anion radical, are essential for various biological functions, including cell survival, cell growth, proliferation and differentiation, and immune response. In the last two decades, it has become apparent that ROS also serve as signaling molecules to regulate biological and physiological processes (Schieber & Chandel, 2014). Thus, the lack of ROS in the immune system may lead to inhibition of the ability to fight invasive pathogens, which may be harmful to aquaculture.

### Microalgae as a food supplement and immunostimulant in global and regional aquaculture

The development of cultures of aquatic organisms, especially fish and shrimp, has shown a tendency to use more intensive production systems (Diana, 2009). Under these conditions, the animals are subjected to different management and environmental conditions (Saboya et al., 2012), the effects of which can be observed in low growth rates (Oliveira et al., 2013), high rates of parasitism (Lizama et al., 2007; Dias et al., 2015), low nutritional levels (Conceição et al., 2009; Forgati et al., 2015) and several changes hematological characteristics (Fries et al., 2013). In addition, this type of cultivation has high mortality rates associated with infection by opportunistic bacteria present in the aquatic flora, which is a direct consequence of infiltration and deterioration of the water quality in the culture (Leonhardt et al., 2011).

Despite this available knowledge, there are still specific gaps that can be considered in this regard, especially in the Amazonian geographical context, due to the need to develop sustainable cultivation in intensive aquaculture. These shortcomings clearly need to be overcome, with the objective of generating technical knowledge capable of overcoming the negative effects of the adverse conditions of this mode of captive rearing (Rodrigues et al., 2009; Moreira, Martins & Farias, 2011).

In recent years, the search for ways to reduce these effects has led to growing scientific interest in the identification of compounds with immunostimulatory activities from microalgae. Immunostimulants are capable of stimulating the body’s immune response, enabling disease control and prevention (Leonhardt et al., 2011; Hoseinifar, Zoheiri & Lazado, 2016; Chagas et al., 2016). Thus, dietary supplementation and immunostimulation have become relevant prophylactic strategies with significant potential for use in aquaculture (Hoseinifar, Zoheiri & Lazado, 2016).

Currently, ≈1,000 t of microalgal biomass are used in world aquaculture, primarily in the feeding of fish fingerlings and juveniles (Priyadarshani & Rath, 2012; Ruffell et al., 2017). This application has demonstrated its influence on the growth rates of cultured organisms, food intake efficiency, better immune responses and effects on the control and treatment of diseases (Adel et al., 2016). Microalgae have also become an integral part of the cultivation of economically important species for aquaculture worldwide (Santos-Ballardo et al., 2015), and these factors increasingly leverage their use in aquaculture processes.

The development of research methods on microalgal bioprospecting and its use in animal feed supplementation represent fundamental advances in the sustainable development and improvement of aquaculture, especially as an alternative to the typical dietary methods employed, valuing the use of products derived from these microorganisms. These advancements represent a notable opportunity for the development of appropriate technologies in aquaculture, especially in the use of native microalgal species from the Amazon, which represents a lack of knowledge regarding this subject, even in the international literature.

Table 2 provides a summary of the primary effects associated with the use of microalgae in the diets of cultured organisms in the last decade, including the minimization of stress, improved health and increased survival of organisms through implications ranging from higher feed intake and digestibility (Fernández-Reiriz, Irisarri & Labarta, 2015; Quang, Pirozzi & Southgate, 2015) to the influence of gene expression of the gastrointestinal tract (Cerezuela, Meseguer & Esteban, 2013). There is still a need for scientific advances in this line of research for the Amazon geographic region, considering the lack of records or studies focused on the biotechnological use of microalgae for regional aquaculture.

**Table 2:** Description of microalgal species, cultured organisms and effects of their administration found in the international literature and by geographical area.

**Microalgae**	**Cultured organisms**	**Effects of administration**	**Geographical area**	**References**
*Arthrospira platensis*	Fish (*Huso huso*)	On growth and high activity of protease and lipase	Iran	Adel et al. (2016)
*Arthrospira platensis*	Shrimp (*Litopenaeus vannamei*)	On final weight, weight gain and survival	Brazil	Gadelha et al. (2013)
*Arthrospira platensis*	Shrimp (*Penaeus merguiensis*)	On increase of phagocytic activity	Singapore	Gadelha et al. (2013)
*Chaetoceros calcitrans*	Shellfish (*Tegillarca granosa*)	On content of fatty acids and sterols	China	Geng et al. (2016)
*Chaetoceros muelleri*	Sandfish (*Holothuria scabra*)	On rate of growth, survival and protein content	Australia	Duy, Francis & Southgate (2017)
*Chaetoceros muelleri*	Sandfish (*Holothuria scabra*)	On the most digestibility	Australia	Quang, Pirozzi & Southgate (2015)
*Chaetoceros muelleri*	Shellfish (*Panopea generosa*)	On the increase of the growth rate and content of fatty acids	Canada	Arney et al. (2015)
*Chaetoceros muelleri*	Shellfish (*Meretrix lusoria*)	On fatty acid profile and number of hemocytes	Taiwan	Chen, Zhao & Qi (2015)
*Chlorella* sp*.*	Fish (*Carassius auratus gibelio*)	On growth and innate immune response	China	Zhang et al. (2014)
*Chlorella vulgaris*	Fish (*Arapaima gigas*)	On increase of immune cells	Brazil	Hoshino et al. (2017)
*Cricosphaera elongata*	Shellfish (*Paracentrotus lividus*)	On survival rate and speed of development	Great Britain	Carboni et al. (2012)
*Diacronema viridis*	Shellfish (*Tegillarca granosa*)	On content of fatty acids and sterols (tendency)	China	Geng et al. (2016)
*Haematococcus pluvialis*	Shrimp (*Litopenaeus vannamei*)	On growth rate and astaxanthin levels	USA	Ju, Deng & Dominy (2012)
*Isochrysis galbana*	Shellfish (*Tegillarca granosa*)	On content of fatty acids and sterols	China	Geng et al. (2016)
*Isochrysis galbana*	calanoid copepod (*Pseudodiaptomus hessei*)	On survival rate and accumulation of fatty acids	South Africa	Siqwepu, Richoux & Vine (2017)
*Isochrysis galbana*	Shellfish (*Meretrix lusoria*)	On lipid fraction and in the increase of lipid peroxidation activity;	Taiwan	Chen, Zhao & Qi (2015)
*Mix of microalgae*	Fish (*Oreochromis niloticus*)	On gastrostatic and enterosomal	Brazil	Moreira, Martins & Farias (2011)
*Nannochloropsis granulata*	Shrimp (*Litopenaeus vannamei*)	On the digestible protein content	Canada	Tibbetts, Yasumaru & Lemos (2017)
*Nannochloropsis granulata*	Fish (*Oncorhynchus mykiss*)	On digestible protein content	Canada	Tibbetts, Yasumaru & Lemos (2017)
*Nannochloropsis oculata*	Shellfish (*Tegillarca granosa*)	On content of fatty acids and sterols	China	Lee et al. (2003)
*Navicula* sp.	Fish (*Sparus aurata*)	On increase of the immune parameters and the leukocyte, peroxidase and complement system activity	Mexico	Reyes-Becerril et al. (2013)
*Navicula* sp*.*	Fish (*Lutjanus peru*)	On increase of total proteins and hemoglobin and in the immune parameters	Mexico	Reyes-Becerril et al. (2014)
*Phaeodactylum tricornutum*	Fish (*Sparus aurata* L.)	On immune parameters and immunostimulatory activities and in the gene expression of the intestinal tract	Spain	Cerezuela et al. (2012) and Quang, Pirozzi & Southgate (2015)
*Porphyridium* sp.	Fish	On antitumor, antiviral, anti-inflammatory and antioxidant activities.	Israel	Siqwepu, Richoux & Vine (2017)
*Rhodomonas lens*	Shellfish (*Mytilus galloprovincialis*)	On the highest intake, digestibility and protein content.	Spain	Santos-Ballardo et al. (2015)
*Rhodomonas salina*	Calanoid copepod (*Pseudodiaptomus hessei*)	On increase of the fecundity rate and accumulation of fatty acids	South Africa	Siqwepu, Richoux & Vine (2017)
*Schizochytrium* sp*.*	Fish (*Salmo salar* L.)	On nutrient retention and fish quality	Norway	Tannin-Spitz et al. (2005)
*Isochrysis galbana*	Sandfish (*Holothuria scabra*)	On rate of growth, survival and protein content	Australia	Geng et al. (2016)
*Tetraselmis chui*	Shellfish (*Meretrix lusoria*)	On fatty acid profile and number of hemocytes	Taiwan	Kousoulaki et al. (2016)
*Tetraselmis chuii*	Fish (*Sparus aurata* L.)	On immune parameters, immunostimulating activities and gene expression of the intestinal tract	Spain	Cerezuela et al. (2012) and Quang, Pirozzi & Southgate (2015)
*Tetraselmis suecia*	Fish (*Sparus aurata*)	On growth performance, nutrient retention and survival rate	Spain	Vizcaíno et al. (2016)
*Tisochrysis lutea*	Shellfish (*Panopea generosa*)	On growth rate and content of fatty acids	Canada	Arney et al. (2015)
*Tisochrysis lutea*	Fish (*Solea senegalenses*)	On high growth rate and levels of lipid absorption and lower daily mortality rate by triacylglycerols increase, phosphocholine and oleic acid	Spain	Chen, Zhao & Qi (2015)
*Tisochrysis lutea*	Fish (*Sparus aurata*)	On increase of docosahexaenoic acid level in the musculature	Spain	Vizcaíno et al. (2016)

In contrast, the use of biomass from microalgae, especially marine types, has been well studied and documented in the international literature for its use in aquaculture (Table 2). These studies show that microalgae, when tested in fish diets, have led to better growth, feed conversion and protein digestibility, resistance to stress and disease, improvement in fish carcass quality and stimulation of early maturation, leading to a shortening of the culture cycle (Román-Padilla et al., 2017).

Similarly, the use of microalgae in association with bacteria for feeding fish (*Sparus aurata*) showed positive effects on the modulation of intestinal gene expression with respect to genes encoding proteins with roles in pro-inflammatory activities, protein transport and digestion and nutrient absorption (Chen, Tseng & Huang, 2015). A similar result was observed for the gene expression of transferrin, the major iron-binding protein in the intestines of fish (*S. aurata*) fed with lyophilized microalgae (Reyes-Becerril et al., 2014), as well as an increase in the number of enterocytes in the intestinal mucosa of fish fed *Tetraselmis suecica* (Vizcaíno et al., 2016).

The microalga *A. platensis* was used as a food supplement for postlarvae of the Nile tilapia (*Oreochromis niloticus*) and had significant effects on its length and final weight (Moreira, Martins & Farias, 2011). Similarly, this microalga promoted the growth performance, spawning rate and coloration when administered at least three times per day in *Maylandia lombardoi* (fish) feed (Karadal, Güroy & Türkmen, 2017).

When incorporated into the feeding of larvae of *Solea senegalensis* (fish), the microalga *Tisochrysis lutea*, conjugated with rotifers, showed a high growth rate compared with other treatments with fish oil and marine lecithin while simultaneously raising the levels of lipid absorption and reducing the rate of daily larval mortality by increasing the levels of triacylglycerols (TAG), phosphocholine (PC) and oleic acid (Román-Padilla et al., 2017).

Fish fed a mixed diet containing the microalgae *Haematococcus pluvialis* and *Ankistrodesmus gracilis* showed higher growth rates, primarily with respect to weight gain (3.4 ± 0.2 g) and the total length of the species (5.0 ± 0.4 cm) for the *Hyphessobrycon eques* diet (Berchielli-Morais, Fernandes & Sipaúba-Tavares, 2016). The combination of different species of microalgae can provide a more balanced diet and can further improve the growth of animals, depending on the diverse nutritional profiles they present (Hemaiswarya et al., 2011).

In addition to fish, shrimp fed *A. platensis* exhibited increased resistance (phagocytic activity) against bacteria (*Vibrio harveyi*, *Escherichia coli*, *Salmonella typhimurium* and *Bacillus subtilis*) in response to the presence of lipopolysaccharides and peptidoglycans (Lee et al., 2003). In addition, shrimp supplemented with 40% lyophilized microalgae presented greater weight gain (3.01 ± 0.43 g) and a better feed conversion rate (2.51 ± 0.43 g) (Gadelha et al., 2013), indicating their effect on the development and survival of cultivated organisms.

The use of microalgae for food supplementation and immunostimulation has positive effects on the development and culture of organisms, supporting their potential application in aquaculture management and control. In addition, these results serve as a reference for assessing diverse influences on the zootechnical, physiological and metabolic performance of these organisms, especially their immunostimulating effects.

This analysis demonstrates the need to perform further studies, which have been rare, in the Amazon region and throughout Brazil (Table 2). This new research may represent an increase, albeit generic, in the potential for biotechnological applications to tropical ecosystems. The use of native microalgae is at least a sustainable alternative for the maintenance of fish stocks, given the emerging and intensive crops that will be present in the future due to demands for food and other human needs (Cunha et al., 2014; Pinaya et al., 2016; Campos-Silva & Peres, 2016; Silva Júnior et al., 2017).

### Bioprospecting studies of microalgae for aquaculture in the Brazilian Amazon

Aquaculture in the Brazilian Amazon is marked by the presence of studies performed over the last several years (2009 to 2017). These studies sought new knowledge regarding aquaculture crop management and performance, especially for fish in the North region (Table 3). The Tambaqui (*Colossoma macropomum*), the most important fish in Brazil (Rodrigues, 2014), has been the focus of research with varied objectives, such as evaluating its productive performance and food intake in the initial phase of cultivation (Sandre et al., 2017), verifying the physiological and pathological changes of the species in response to parasitism (Jerônimo et al., 2017), supporting the effects on reproductive induction (Martins et al., 2017), determining factors for genetic improvement and gene expression (Gomes et al., 2017; Perazza et al., 2017) and evaluating side effects to antiparasitic, such as mebendazole (Chagas et al., 2016).

**Table 3:** Description of studies performed exclusively in the Amazon region, with a focus on the aquaculture of endogenous species.

**Organism studied**	**Purpose of the study**	**References**
Shrimp (*Macrobrachium amazonicum*)	To evaluate the antimicrobial action of *Moringa oleifera* against *Vibrio* spp. in shrimp farming	Brilhante et al. (2015)
Brycon (*Brycon amazonicus)*	To evaluate the effects of secondary metabolites of higher plants on dietary supplementation	Ribeiro et al. (2016)
Arapaima (*Arapaima gigas*)	To evaluate parasite infestation	Delgado, Delgado & Orbe (2013)
Arapaima (*Arapaima gigas*)	To evaluate parasite infestation	Araújo et al. (2009)
Tambaqui hybrid (*Colossoma macropomum*×*Piaractus mesopotamicus*)	To study parasitic fauna	Silva et al. (2013)
Tambaqui (*Colossoma macropomum*)	To evaluate their productive performance and food intake in the initial phase of cultivation	Sandre et al. (2017)
Tambaqui (*Colossoma macropomum*)	To evaluate physiological and pathological changes in response to parasitism	Jerônimo et al. (2017)
Tambaqui (*Colossoma macropomum*)	To evaluate reproductive induction	Martins et al. (2017)
Tambaqui (*Colossoma macropomum*)	To evaluate genetic improvement and gene expression	Perazza et al. (2017)
Tambaqui (*Colossoma macropomum*)	To assess side effects to antibiotics	Chagas et al. (2016)
Tambaqui hybrid (*C. macropomum*×*P. brachypomus*)	To study the physiological and performance effects on diets with Brazil nuts	Santos de et al. (2010)

Similarly, studies of parasitic fauna of *Tambaqui hybrids* (*Colossoma macropomum* × *Piaractus brachypomus* and *Colossoma macropomum* × *Piaractus mesopotamicus*) under cultivation conditions were also performed (Silva et al., 2013; Dias et al., 2015; Pinheiro et al., 2015; Winckler et al., 2015), as were studies of *Arapaima* (*Arapaima gigas*) specimens grown in Amazonia Peruvian (Delgado, Delgado & Orbe, 2013; Mathews et al., 2014) and in the Brazilian Amazon (Araújo et al., 2009; Marinho et al., 2013; Santos, Da Silva & Moravec, 2017), including those performed in the extreme north of Brazil (Amapá State) (Dias et al., 2015; Hoshino et al., 2017). In addition, research on the use of secondary metabolites of higher plants has also been undertaken to identify important factors for the better management and maintenance of aquaculture processes in the region (Brilhante et al., 2015; Barbas et al., 2016; Barbas et al., 2017; Ribeiro et al., 2016; Soares et al., 2017; Dutra et al., 2017).

Although these investigations were conducted in a relatively short period of time (2009–2017), the complexity of the processes involved in aquaculture in the Brazilian Amazon is remarkable. Thus, there is an initial need to improve the levels of basic knowledge of these processes and management to avoid the deleterious effects of cultivated organisms, both in their natural habitats (semi-intensive cultivation) and under controlled conditions (intensive cultivation).

In the Amazon, bioprospecting research has shown relative improvements in the discovery and extraction of secondary metabolites from higher plants (Brilhante et al., 2015; Ribeiro et al., 2016; Barbas et al., 2017). However, bioprospecting of microalgae for aquaculture is completely incipient or unknown, although these shortcomings demonstrate a great deal of potential for new and promising studies regarding the use of microalgal biodiversity, involving both the discovery of species as well as their effective prospective use in economic, social and environmental sectors (Silveira Júnior et al., 2015).

Among the few studies conducted in the Amazon region for microalgal bioprospecting, some have used strains that were imported from other regions of Brazil and worldwide (Costa, Koening & Pereira, 2005) or were conjugated to probiotics of commercial origin (Hoshino et al., 2017), resulting in an extensive knowledge gap on the potential use of microalgae from the Amazon region itself.

These gaps are also derived from the history of the productive and biotechnological sector in Brazil. For example, microalgal cultivation began to be developed in Brazil to meet the needs of aquaculture and environmental sanitation less than two decades ago (Lourenço & Vieira, 2004). However, ten years ago, there were approximately 40 research centers (institutes and universities) in which macro- and microalgal crops, including cyanobacteria, were maintained, some with considerable numbers of isolates (approximately 150 strains) (Brasil, Silva & Siqueira, 2017). However, this approach has led to a delay in studies involving biotechnology and bioprospecting of microalgae in Brazil, especially for important purposes such as human nutrition and the production of drugs or biofuels (García, Vicente & Galán, 2017).

Thus, research on microalgal cultivation in Brazil began to develop more rapidly at the beginning of the present century, remaining sparse in the last ten years and primarily concentrated in the South and Southeast regions of the country (Table 4), which do not include the Amazon region (Müller, Rodriguez-Amaya & Lourenço, 2003; Bertoldi, Sant’Anna & Oliveira, 2008; Ohse et al., 2009; Borges-Campos, Barbarino & Lourenço, 2010; Bastos & Bonini, 2017). This same pattern of research has been observed in other lines of research involving microalgae, such as taxonomy and ecology, which have had almost “neutral” and no direct effects on the development of more applied research for the region (Silveira Júnior et al., 2015).

**Table 4:** Description of experimental studies published in scientific journals involving the cultivation and/or bioprospecting of microalgae for the Brazilian territory and their application potential.

**Organism studied**	**Objectives of the study**	**Potential for application**	**Brazilian geographic region**	**References**
*Aphanothece microscopica; Chlorella vulgaris*	To evaluate the mixotrophic culture of microalgae in medium supplemented with potassium acetate.	• Optimization of microalgal cultivation processes • Production of biodiesel • Feeding animals	Southeast	Bastos & Bonini (2017)
*Ankistrodesmus fusiformis; Chlorella vulgaris; Desmodesmus spinosus*	To determine the influence on the growth and accumulation of total lipids of three species of microalgae Chlorophyceae with potential for the production of biodiesel on a commercial scale.	• Optimization of microalgal cultivation processes • Lipid synthesis • Production of biodiesel	Southeast	Martins & Fernandes (2016)
*Arthrospira platensis*	To evaluate adaptation of the cultured cyanobacteria to swine effluent and to determine the ideal dilution of effluent to obtain the maximum biomass production and removal of Chemical Oxygen Demand (COD), ammonia and phosphorus from the effluent by the cyanobacteria	• Mitigation of environmental impacts by effluents. • Alternative to swine wastewater treatment • Feed supplements in fish farming • Use of biomass as fertilizer.	South	Mezzomo et al. (2010)
*Arthrospira platensis*	To evaluate the growth of *Spirulina platensis* in culture medium supplemented with liquid molasses (MEL) and powder molasses (MEP).	• Optimization of microalgal cultivation processes • Potential use in human food	South	Andrade & Costa (2008)
*Chaetoceros muelleri, Isochrysis galbana, Isochrysis* sp., *Nannochloropsis oculata, Phaeodactylum tricornutum, Tetraselmis suecica, Tetraselmis chuii, Thalassiosira pseudonana and Thalassiosira fluviatilis*	To evaluate the productivity and the carbon content, hydrogen, nitrogen and protein	• Production of biodiesel • Reduction of CO_2_ sequestration (Environmental Services and mitigating environmental impacts) and Environmental Recovery	South	Ohse et al. (2009)
*Chlorella* sp.	To determine the potential of cultivation of the microalga *Chlorella* sp. in culture medium composed of wastewater	• Production of biodiesel • Optimization of microalgal cultivation processes	Northeast	Vieira et al. (2014)
*Chlorella* sp. and *Scenedesmus* sp.	To evaluate the rheological behavior of microalgae in different concentrations of biomass	• Production of biodiesel • Optimization of microalgal cultivation processes	Southeast	Santos de et al. (2013)
*Chlorella vulgaris*	To evaluate the composition of mineral salts and the contents of chlorophyll **a** and **b** present in the microalga Chlorella vulgaris cultivated in residual hydroponic solution.	• Development of nutritional supplements • Optimization of microalgal cultivation processes	South	Bertoldi, Sant’Anna & Oliveira (2008)
*Chlorella vulgaris*	To evaluate the growth, biomass productivity and biochemical production and composition of microalga in semi-continuous cultures using different growth media	• Optimization of the microalgal cultivation process • Food supplements in fish farming	Southeast	Chia, Lombardi & Melao (2013)
*Chlorella vulgaris* associated with yeasts	To evaluate the hematological, biochemical and physiological characteristics of fish supplemented with diet including microalgae	• Food supplementation in fish farming	North (Amazon)	Hoshino et al. (2017)
*Gyrodinium corsicum* and *Rhodomonas baltica*	To evaluate the insertion of microalgae in zooplankton feeding	• Reduction of potentially toxic microalgae blooms in natural environments	North (Amazon)	Costa, Koening & Pereira (2005)
*Monoraphidium contortum*	To determine the secondary metabolites and to evaluate the cytotoxicity activity in *Artemia saline* and the antioxidant activity of the crude methanolic extract of cyanobacteria	• Use of secondary metabolites for biotechnology and related fields	North (Amazon)	Tanaka et al. (1997)
*Monoraphidium contortum, Chlorella vulgaris and Desmodesmus quadricauda*	To evaluate the influence of temperature and nutrients on the lipid contents of cultured freshwater microalgal species	• Optimization of the microalgal cultivation process aiming at higher lipid production in the culture and its biotechnological use	South	Bohnenberger & Crosseti (2014)
*Phormidium* sp.	To evaluate the production of third-generation biodiesel	• Production of biodiesel • Use of bioactive compounds for biotechnological purposes	Southeast	Francisco et al. (2015)
*Synechocystis pevalekii*	To determine the composition of carotenoids of the species studied, contributing to the knowledge about Brazilian natural resources	• Use in the textile industry • Use in the pharmaceutical industry	Southeast	Müller, Rodriguez-Amaya & Lourenço (2003)

Different growth media (LC Oligo, WC and CHU) in semicontinuous cultures were evaluated for the species *Chlorella vulgaris*, with the highest growth rate (0.84 day^−1^), cell density (2.74 × 10^6^ cell m^−1^) and yield (≈16 pg cell^−1^) observed for LC Oligo medium. In contrast, the highest contents of lipids (≈0.9 pg cell^−1^), carbohydrates (≈4 pg cell^−1^) and proteins (≈6 pg cell^−1^) were observed for the CHU medium (Chia, Lombardi & Melao, 2013). These studies confirm that the greater proportion of lipids obtained can present positive effects for the growth and immunological and physiological performance of herbivores that are fed *C. vulgaris*, such as some fish and crustaceans. These findings represent advances in the local subsidiary processes regarding the use of microalgae in the diets of organisms in aquaculture.

Similarly, the manipulation of temperature and nutrients was observed to influence the lipid contents produced in strains of microalgae, including *C. vulgaris*, *Desmodesmus quadricauda*, *Monoraphidium contortum* and *Microcystis aeruginosa*, indicating a strategy for increasing biomass and a higher lipid productivity profiles, and consequently, a greater possibility of their use for different purposes (Bohnenberger & Crosseti, 2014).

Dinoflagellates were used to feed zooplankton to assess the degree of toxicity presented by microalgal species (Costa, Koening & Pereira, 2005). For aquaculture, this strategy is essential to understand the pattern of toxin bioaccumulation in the aquatic food chain and the input (zooplankton) that can be used in the diet of fish and crustaceans in culture systems.

In this scenario, 54.16% of the work performed in the last several years in Brazil (Table 4) has the potential for application to promote improvements of microalgal cultivation processes (29.16%) and their potential applicability in the production of biodiesel (25.0%). On the other hand, 16.6% considered the potential use of the obtained data in aquaculture. The remaining studies included applications in human nutrition (8.33%), the provision of environmental services (8.33%), the mitigation of environmental impacts (8.33%) and inputs for fertilizer production (4.16%).

The Amazon has highly favorable environmental and ecological conditions for the development of microalgal cultivation. These conditions include the availability of high intensity solar radiation by area and time (Marques et al., 2012), seasonal thermal stability and areas with gentle slopes and hydromorphic clay soils for the construction of lakes or culture tanks combined with abundant water availability (Azeredo, 2012). However, there are still several obstacles to the prospective use of biodiversity associated with regional aquaculture, especially to those focused on industrial-scale processes, a later stage to the basic bioprospecting of microalgae.

Therefore, it is noted that additional efforts are needed to increase the number of cultivation and bioprospecting studies of these microorganisms throughout Brazil (Tables 2 and 4). This approach would likely minimize the regional asymmetries of the country’s productive/biotechnology sector in the use of microalgae and its multiple purposes (including aquaculture) and would also enable technical and scientific advancements in strategic zones worldwide, such as the Brazilian Amazon.

## Conclusions

An understanding of the synthesis of bioactive compounds of microalgae (Table 1) and their technological-economic potential is important for their various purposes and applications, including bioenergy and aquaculture.

The numerous bioprospective studies on microalgae described in the literature suggest that their applications in food supplementation and immunostimulation of aquatic organisms are potentially strategic and essential to promote the sustainable (economic and technological) maintenance of aquaculture.

In addition, some studies have sought to improve the microalgal production to make possible the best bioprospective use this microorganism in the most diverse areas. This research has allowed more direct applications of microalgae in aquaculture, especially in food supplementation and immunostimulation.

The results of studies (Table 2) in this context have allowed us to confirm that microalgae have the potential to improve aquaculture production, with significant effects observed on the development of aquaculture worldwide that can transcend local and regional processes.

In a global overview, this review presents a contribution to the literature on the advantages and limitations of bioprospecting applications from microalgae, revealing an excellent perspective for biotechnology and prospecting for the use of this input.

For Brazil, small advances have been made in the development of technologies used for microalgae involving the selection, cultivation and production of biomass of variable species and for different purposes. The few studies in the literature on the bioprospection of microalgae in the Amazon region indicate their potential applicability as a strategic alternative for the development of regional aquaculture.

Finally, the use of local preexisting knowledge, although lacking scientific rigor, can and should still provide support for the conservation of natural fish stocks using microalgal biomass naturally available in the region.

## Additional Information and Declarations

### Data Availability

The following information was supplied regarding data availability: The research in this article did not generate any data or code, as this article is a literature review.
